# New Section: Forensic Diagnostics

**DOI:** 10.3390/diagnostics16101417

**Published:** 2026-05-07

**Authors:** Andreas Kjaer

**Affiliations:** 1 Cluster for Molecular Imaging, Department of Biomedical Sciences, University of Copenhagen, 2200 Copenhagen, Denmark; akjaer@sund.ku.dk; 2 Department of Clinical Physiology and Nuclear Medicine, Copenhagen University Hospital, Rigshospitalet, 2100 Copenhagen, Denmark

It is with pleasure that we announce the launch of a new section of *Diagnostics*, named “Forensic Diagnostics”. This section will focus on manuscripts covering the following topics: post-mortem imaging and virtopsy; forensic pathology and chemical analysis; molecular diagnostics for identification and kinship analysis; diagnostic techniques for trauma and injury assessment; and advances in analytical technologies applied to forensic science. All submissions to this section must align with the overall scope of *Diagnostics*.

We hope that the “Forensic Diagnostics” section will attract high-quality manuscripts within this specialized field and provide clearer guidance for authors regarding the appropriate placement of their work. The Editors and the Editorial Office will, as always, assist in allocating manuscripts to the section that best suits their subject matter.

We welcome researchers working in forensic diagnostics to submit their work to this new section. In addition, we invite interested scholars to contribute to the development of this section by proposing Special Issues or applying to join the Editorial Board. We look forward to your valuable contributions in making “Forensic Diagnostics” a vibrant and impactful platform within the journal.

